# Construction of Genealogical Knowledge Graphs From Obituaries: Multitask Neural Network Extraction System

**DOI:** 10.2196/25670

**Published:** 2021-08-04

**Authors:** Kai He, Lixia Yao, JiaWei Zhang, Yufei Li, Chen Li

**Affiliations:** 1 School of Computer Science and Technology Xi’an Jiaotong University Xi’an China; 2 National Engineering Lab for Big Data Analytics Xi’an Jiaotong University Xi’an China; 3 Shanxi Province Key Laboratory of Satellite and Terrestrial Network Technology Research and Development Xian China; 4 Department of Health Sciences Research Mayo Clinic Rochester, MN United States

**Keywords:** genealogical knowledge graph, EHR, information extraction, genealogy, neural network

## Abstract

**Background:**

Genealogical information, such as that found in family trees, is imperative for biomedical research such as disease heritability and risk prediction. Researchers have used policyholder and their dependent information in medical claims data and emergency contacts in electronic health records (EHRs) to infer family relationships at a large scale. We have previously demonstrated that online obituaries can be a novel data source for building more complete and accurate family trees.

**Objective:**

Aiming at supplementing EHR data with family relationships for biomedical research, we built an end-to-end information extraction system using a multitask-based artificial neural network model to construct genealogical knowledge graphs (GKGs) from online obituaries. GKGs are enriched family trees with detailed information including age, gender, death and birth dates, and residence.

**Methods:**

Built on a predefined family relationship map consisting of 4 types of entities (eg, people’s name, residence, birth date, and death date) and 71 types of relationships, we curated a corpus containing 1700 online obituaries from the metropolitan area of Minneapolis and St Paul in Minnesota. We also adopted data augmentation technology to generate additional synthetic data to alleviate the issue of data scarcity for rare family relationships. A multitask-based artificial neural network model was then built to simultaneously detect names, extract relationships between them, and assign attributes (eg, birth dates and death dates, residence, age, and gender) to each individual. In the end, we assemble related GKGs into larger ones by identifying people appearing in multiple obituaries.

**Results:**

Our system achieved satisfying precision (94.79%), recall (91.45%), and F-1 measures (93.09%) on 10-fold cross-validation. We also constructed 12,407 GKGs, with the largest one made up of 4 generations and 30 people.

**Conclusions:**

In this work, we discussed the meaning of GKGs for biomedical research, presented a new version of a corpus with a predefined family relationship map and augmented training data, and proposed a multitask deep neural system to construct and assemble GKGs. The results show our system can extract and demonstrate the potential of enriching EHR data for more genetic research. We share the source codes and system with the entire scientific community on GitHub without the corpus for privacy protection.

## Introduction

Anthropologists often use oral interviews, historical records, genetic analysis, and other means to obtain genealogical information and draw family trees. When combined with a detailed medical history and social and economic relationships, family trees are considered the x-ray of the family and have been used by clinicians to assess disease risk, suggest treatments, recommend changes in diet and other lifestyle habits, and determine a diagnosis. In the United States, the Medicare Access and CHIP Reauthorization Act of 2015 [1] and Meaningful Use program [2] have incentivized the growing adoption of electronic health records (EHR) with the goal to improve the quality of health care delivery systems. Consequently, a vast amount of EHR data has become available for research purposes in the past decade. However, most EHR systems today do not capture the family relationships between patients by design. Nor do they capture the death information unless patients die in the health care system or the EHR system is linked to external death registries. Constructing family trees for patients becomes an urgent need to unlock the full potential of EHR data in understanding disease and trait heritability, evaluating individuals’ health risks, and exploring environmental effects on human health.

Early exploratory works have combined EHR data and family trees for biomedical research. For instance, Mayer et al [3] used twin or multiple relationships to assess the concordance rates for muscular dystrophy and fragile X syndrome in the twin cohort. Schalkwyk et al [4] conducted interviews with family members to build 3-generation family trees with medical chronologies and demonstrated their use in deciding the services required for the psychological well-being of all family members. Wang et al [5] combined diagnosis codes and dependent coverage under medical plans to estimate the heritability and familial environmental patterns of 149 diseases and inferred the genetic and environmental correlations between 29 complex diseases [5]. Similarly, Polubriaginof et al [6] built more than 595,000 family trees from emergency contact information in a large EHR system and then estimated the heritability of 500 traits.

Constructing high-quality large family trees has been challenging. Historically, only famous politicians, philosophers, scientists, religious groups, or royal families were tracked elaborately by genealogists. For such reason, large databases of family trees rarely existed, despite their research value. Recently, a few studies automated family tree collection using innovative informatics approaches. For instance, Mayer and colleagues [3] used shared dates of birth and last names, in addition to home addresses, billing accounts, and keywords of “twin” and “triplet” in unstructured clinical notes to identify a cohort of 19,226 twins or multiples in an extensive health care delivery system. Wang et al [5] inferred 128,989 nuclear families from a large medical claims database covering one-third of the US population based on dependent coverage. Polubriaginof et al [6] used the emergency contact information of 3,550,598 patients from three large EHR systems in New York City to build 595,000 pedigrees. However, these indirect sources, like dependent coverage and emergency contact, have inherent limitations for inferring genealogical information: they do not differentiate biological from nonbiological relationships and they cover only limited types and numbers of family relationships. More specifically, medical insurance in the United States is limited to a beneficiary’s spouse and dependents up to age 26 years. Most patients only provided one or two emergency contacts rather than their whole families in their medical records. Missing relationships could be substantial.

Inspired by the work of Tourassi et al [7] and Yoon et al [8], we began to explore online obituaries as a novel data source for the automatic extraction of genealogical information. Obituaries generally cover many more family members with more detailed and accurate descriptions of their family relationships. Nowadays, local newspaper and funeral service companies often publish obituaries on internet, making the cost of obtaining obituaries minimal. In our previous work, we developed and evaluated a new method of name entity recognition (NER) for extracting family members’ names and relation classifications (RCs) for classing the pairs of names among family members mentioned in online obituaries [9]. In this work, we advanced our previous work in the following 5 aspects: (1) for the NER task, we processed more entity types, including people’s name, age, residence, and dates of birth and death; (2) for RC, we also matched residence entity and related people (in the previous work, we only extracted the family relationships among people entity); (3) we parsed two kinds of special language patterns in obituaries, last name distributive and name with parentheses; (4) all the triplets of family relationships were integrated to build the enriched family trees with additional rule-based inference; and (5) in terms of training data, we normalized the family relationships (see details in Data section).

Traditionally, NER and RC were considered two separate tasks for information extraction. NER sought to extract named entities mentioned in unstructured text into predefined categories, whereas RC classified the relations between those extracted entity mentions. Researchers built natural language processing (NLP) pipelines with multiple modules to accomplish specific tasks. However, such modular separation suffered from 3 major issues leading to suboptimal results: (1) errors from the NER propagated to RC, (2) it was computationally redundant and time-consuming as the system had to pair up every two named entities to classify their relations, and (3) the pipeline model could not take full advantage of the knowledge inhabitant in the relationships of 2 or more named entities. For instance, if the system detected a *live in* relationship between two named entities in obituaries, the first entity is likely to be a person’s name and the second entity is likely to be a location.

Thus, we look at multitask models that can simultaneously handle multiple related tasks and optimize their learning abilities by sharing the knowledge learned in all or some of the tasks [10]. In 2008, Collobert [11] introduced a single neural network architecture that solved NLP tasks such as part-of-speech tagging, chunking, named entity recognition, semantic role identification, and semantically similar word grouping using one language model. Recently, there are 3 prevailing solutions for multitask NLP models. The most popular solution establishes a common neural network presentation space for all tasks followed by task-specific classifiers [12,13]. The second proposes novel neural network architecture for multiple tasks. Sun et al [14] used graph convolutional networks to enhance interaction between entity and relation. Bhatia et al [15] proposed a hierarchical encoder-decoder NER model and adopt a shared encoder followed by separate decoders for NER and RC tasks. The third focuses on customized tagging schema. For instance, Zheng et al [16] proposed a novel tagging schema for long short-term memory (LSTM) models that simultaneously identified named entities and extracted relationships in a corpus of New York Times news. In addition, Dixit et al [17] introduced a span-level solution to handle NER and RC together. Zheng et al [18] introduced a hierarchical solution that combines an encoder-decoder LSTM module for NER with a convolution neural network for RC.

In this work, we first updated our annotated corpus by defining a family relationship map to normalize various family relations (see details in Data section). We also used data augmentation technology to generate more synthetic data (sentences), in order to address the imbalanced training data issue and boost the performance on rare classes [19]. After that, we proposed an end-to-end information extraction system based on a multitasking solution. The end-to-end system included a knowledge inference layer for gender inference based on name and relationship mentioning. In the end, we constructed family trees centered on the deceased. These family trees contained many family members with detailed information, including age, gender, death date, birth date, and residence. We named such enriched family trees genealogical knowledge graphs (GKGs). These GKGs could be linked to external EHR data in Minnesota by personally identifiable information (PII), in a similar way as Sauver et al [20] did. We empirically estimated the upper bound of the mapping precision could be around 80% to 90%. It would significantly enhance the power of EHR data to study disease and trait heritability, evaluate an individual’s health risks, and explore environmental effects on the human health.

## Methods

### Data

We collected 12,407 obituaries published from October 2008 to September 2018 from 3 funeral services websites and 1 local newspaper in the Twin Cities area, metropolitan Minneapolis–Saint Paul. Our data sources were limited to openly available obituaries. Considering the PII embedded in online obituaries, we decided to take a cautious and conservative position in our work by marking up the last name of any real people with the symbol XX (see more details on privacy protection in the Discussion section). After data cleaning, we randomly sampled 1700 obituaries for annotation. We developed the annotation guideline and trained 3 annotators to annotate each of the 1700 obituaries independently. The interannotator agreement measured by F-1 was 82.80% [9]. Table 1 shows the summary statistics of the annotated corpus. There were two unique language patterns in obituaries, namely last name distributive and name with parentheses (see Table 2 for examples). These patterns might be due to the word limitation when the family paid for publishing an obituary in printed newspapers. They required special treatment, as described in the next session of end-to-end system.

**Table 1:** Summary statistics of the annotated corpus.^a^

Corpus	Count	Deceased person	Count	Special language patterns	Count
Sentences	28,317	Full name	1551	Last name distributive	4954
Names	27,108	Age	1379	Name with parentheses	7504
Family relationship	25,557	Death date	1557	Spouse’s name	5993
Residence	7161	Birth date	1368	Previous last name	1511
Name-residence pair	7954	—^b^	—	—	—

^a^All counts are the number of occurrences except for the full name of the deceased. Considering all obituaries have structured metadata giving the full names of the deceased more precisely, we only annotate and extract the first-time mention of a full name of the deceased in an obituary. Spouse’s name and previous last name are 2 categories in the content name with parentheses.

^b^Not applicable.

**Table 2:** Examples of unique language patterns in obituaries.

Language pattern		Example	Explanation
Last name distributive		He is survived by grandsons Addison and Owen XX.	XX is also the last name for Addison.
**Names with parentheses**			
	Previous last name	Anne was born March 20, 1952, to William and Isabel (Starr) XX.	Starr is the maiden name for Isabel XX.
	Spouse’s name	Survived by her sons, Dale (Mary) and Bruce (Diana).	Dale’s wife is Mary, and Bruce’s wife is Diana.

**Figure 1:**
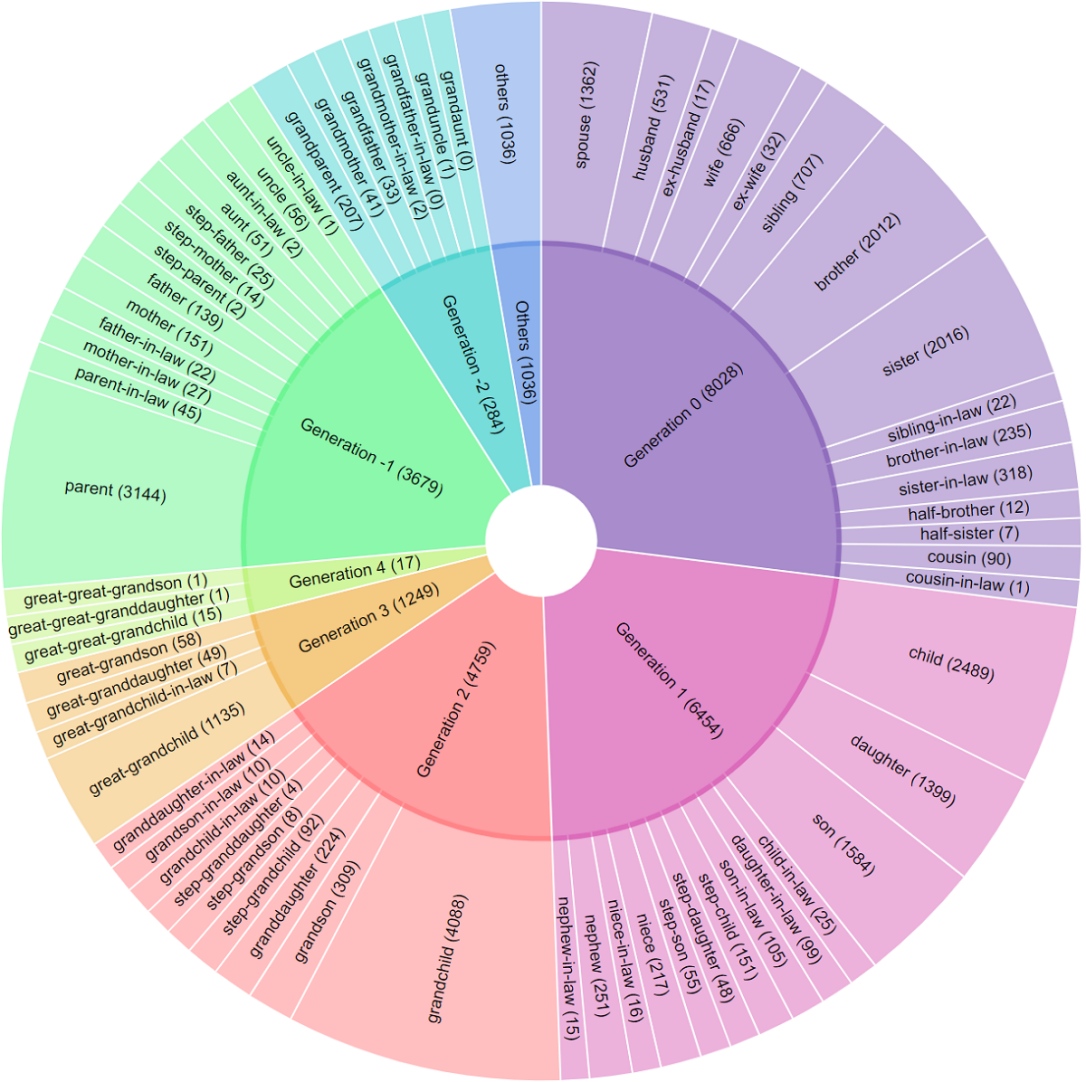
Family relationship map in the obituary corpus.

In this work, we made two improvements in the corpus annotations. First, we created a family relationship map that normalized various family relationship mentions to 71 family relationship groups. For example, there were many mentions of “born to (name),” “daughter of (name),” and “son of (name)” in obituaries, which were equivalent to express the parent of the deceased. We grouped them into the “parent” relation. Similarly, we treated “married to” the same way as the “spouse (of)” relation. Figure 1 shows the family relationship map, consisting of 8 generations and 71 normalized family relationships. The numbers in parentheses were the number of occurrences of a specific family relationship in our corpus.

It was observed that some family relationships, such as granduncle, uncle-in-law, and half-sister had small numbers of cases that was not sufficient to train a high-performance neural network model. Therefore, we used data augmentation technology [19] to expand the corpus and alleviate the imbalanced data issue. We first introduced *w_i_*, the weight of relation *i*:


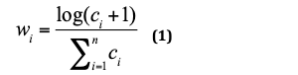


Where *c_i_* stood for the count of annotated sentences with relation *i*, and *n* was 71, the count of all family relationship groups defined in the family relationship map. For each family relationship *i*, the number of training sentences to be generated, *g_i_*_,_ was computed as follows:


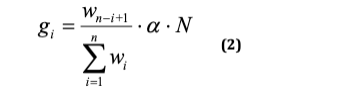


Where *N* was the total number of all human annotated sentences and 𝛼 was the user-defined ratio for data augmentation. Essentially we generated more synthetic sentences to ensure each family relationship had no less than 200 examples, with the constraint that the count ratios of all family relationships remain as close as possible to those in the original training data.

After deciding *g_i_*, 2 steps were performed to generate extra sentences. First, we randomly chose *g_i_* sentences from the raw corpus and replaced one of the raw family relationship tokens in these sentences with relationship word *i*. Second, we randomly chose one of the following operations introduced by Wei and Zou [19] to generate the final augmented sentences:

- Synonym replacement: randomly replace *n* non–stop words with their synonyms in the sentence
- Random insertion: randomly insert a word’s synonym before or after the chosen non–stop word in the sentence
- Random swap: randomly swap 2 non–stop words in the sentence
- Random deletion: randomly remove a non–stop word in the sentence

The 4 entity types of interest in this work, name, residence, birth date and death date, are exempt from the changes. It should also be noted that the generated sentences could not be guaranteed to be grammatically and semantically correct. However, for neural network models, such sentences, when created with appropriate *α*, were demonstrated to improve models’ generalizability as noisy training data.

### End-to-End System

**Figure 2:**
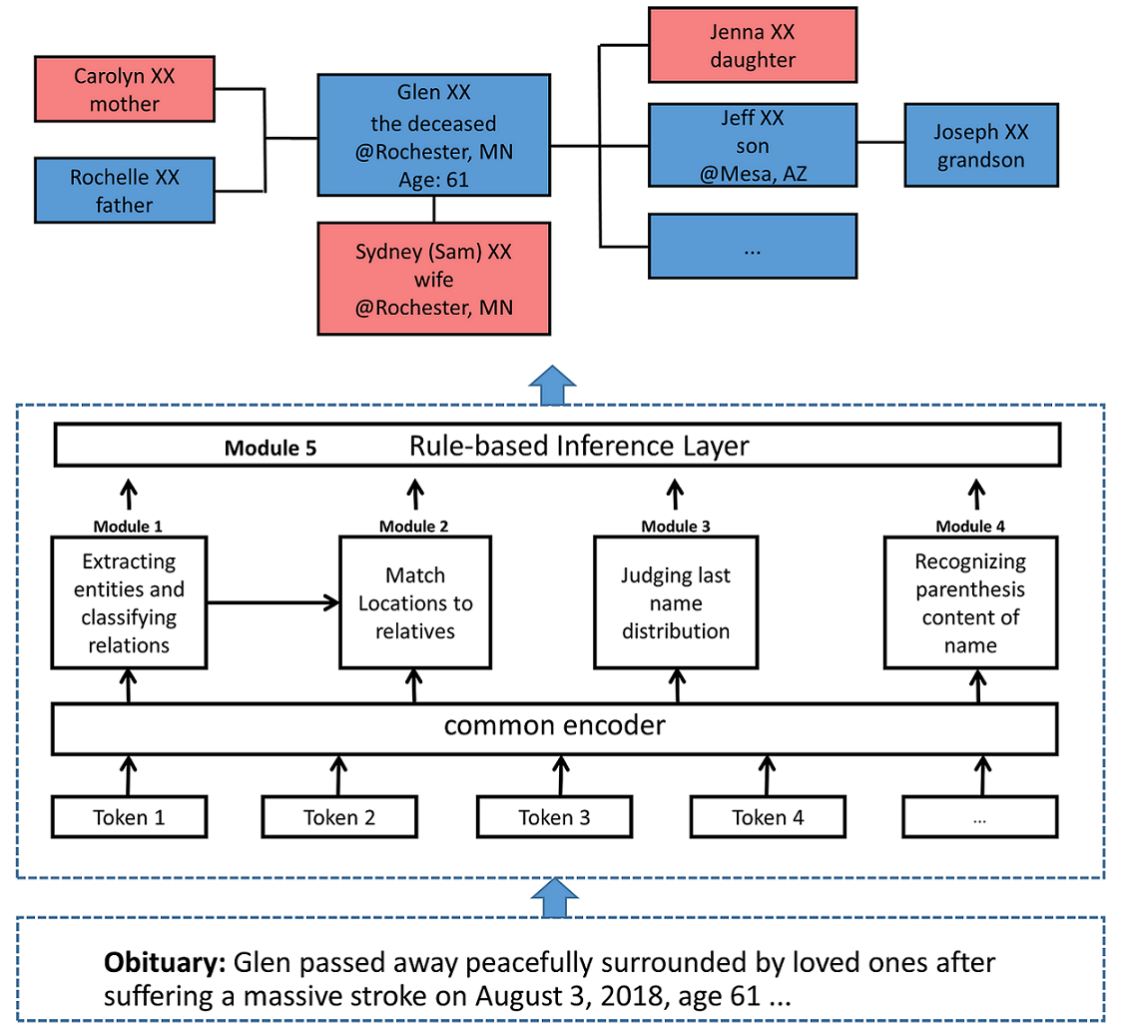
End-to-end extraction system to parse obituaries and generate genealogical knowledge graphs.

Figure 2 illustrates our end-to-end system. It took a list of segmented sentences in an obituary as the input and generated a GKG centered around a deceased person. Its core was a multitask system that combined common parameter sharing across different modules and custom tagging schemes. The multitask solution promised better performance, as it used more supervision information and understood data from different views [21]. The 4 modules were (1) named entity recognition and relation classification through a joint training model and customized tagging scheme, (2) matching locations to people’s name, (3) a parser for resolving last name distributive, and (4) a parser for resolving names with parentheses. These 4 modules shared the same model parameters, as they were trained jointly using one common weighted loss function. Among these modules, module 2 needed the extracted names and locations from module 1 as inputs. Module 5 was added as an independent rule-based layer for gender inference and age, date of death, and birth inference. Eventually, the results of these modules were combined to construct the GKGs.

#### Module 1: Joint NER and RC

**Figure 3:**
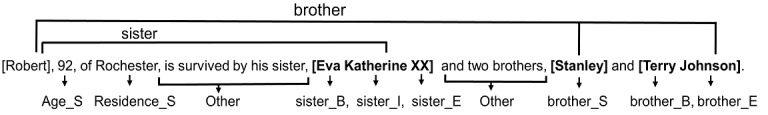
Tagging scheme for simultaneously extracting entities and kinship. S: single; B: begin; I: inside; E: end.

This module aimed to extract family members’ names, relationships, and additional attributes of people (residence, age, death date, birth date). Gender was usually not explicitly mentioned in the obituaries, so we inferred the gender in module 5. We adopted a customized tagging scheme (shown in Figure 3) when annotating the training data. Each tag consisted of 2 parts. The first part indicated the type of an entity, and the second part illustrated the position of the word in the entity. As shown in Figure 3, “sister_B,” “sister_I,” “sister_E,” and “Age_S” indicated the beginning, the inside, and the end of a sister entity and a single-word entity of age, respectively. In the system, the deceased was the default baseline entity for all family relationship triplets. In the sentence shown in Figure 3, for example, “Robert” was the name of the deceased person (we knew it from the obituary metadata and the context of the entire obituary). After annotation, we obtained three triplets (Robert, sister, Eva Katherine XX), (Robert, brother, Stanley), and (Robert, brother, Terry XX). The calculating process was as follows:


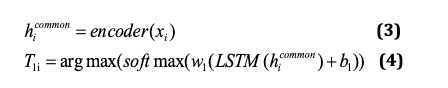


For each input token *x_i_*, we used BERT [22] as a common encoder to obtain each hidden representation *h_i_^common^*. Then *h_i_^common^* were sent into one LSTM classifier to obtain each tag *T_1i_* ∈ *T*_1_, where *T_1_* was the result set of module 1, and *w_1_* and *b_1_* were parameters for training.

#### Module 2: Matching Locations to People

After identifying the residence entities (eg, Rochester in Figure 3), we need to match them with specific people. To do so, we used 3 inputs, all extracted names *T*_1_^name^ ∈ *T*_1_, all extracted residences *T*_1_^residence^ ∈ *T*_1_, and common representation *h_i_^common^*. This module followed by a co-reference solution [23]. We defined the process as follows:

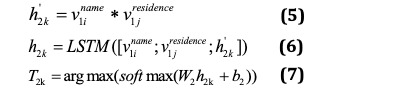



where *v*_1i_*^name^* denoted the vector of one name entity *t*_1_*_i_*^name^ ∈ *T*_1_*^name^*, *v*_1_*_j_^residence^* denoted the vector of one residence entity *t*_1_*_j_*^residence^ ∈ *T*_1_*^residence^*, [] denoted concatenate, * denoted dot product, *w_2_* and *b_2_* were one linear layer parameters for training, *T*_2k_ ∈ *T*_2_ was the matching result for each pair of name and residence, and *T_2_* was the final selected pair of name and residence.

#### Module 3: Judging Last Name Distributive

We identified 2 special language patterns in obituaries, last name distributive and names with parentheses, as shown in Table 2. Resolving these language patterns was helpful for extracting and constructing high-quality GKGs. The task of module 3 was to decide for each token in an input sentence if the last name distributive existed by assigning each token with a binary tag of yes OR no. When we cotrained module 3 with other modules, these tags would concatenate with other modules’ tags for joint training. In the sentence in Figure 3, for example, “Stanley” and “Terry” shared the same last name of “Johnson.” Therefore, in module 3, “Stanley” was assigned a label “brother_S_yes” and “Terry Johnson” was given 2 tags “brother_B_yes” and “brother_E_yes.” This way, the module would extract 2 full names, *Stanley Johnson* and *Terry Johnson*, instead of *Stanley* and *Terry Johnson*. The detailed computing process was as follows:


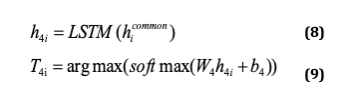


where *w_4_* and *b_4_* were parameters for training, *T*_4i_ ∈ *T*_4_ was the result for each name, and *T_4_* was the result set of module 3.

#### Module 4: Recognizing Names With Parentheses

Module 4 was a 3-class classifier to determine whether there was a parenthesis in a name, and if so, whether it referred to a previous last name or the name of spouse. The computing process was the same as module 3, which took the input of *h_i_^common^* and output the tags of 3 classes (“no parenthesis,” “previous last name,” and “spouse’s name”).

#### Module 5: Rule-Based Inference Layer

This module aimed to infer age, death date, and birth date for the deceased and gender for both the deceased and their family members. First, if an obituary mentioned any 2 attributes out of age, birth date, and death date for the deceased, we calculated the third one. Second, we used both family relationship keyword and name to infer gender. If a family relationship keyword (eg, son, daughter, nephew) suggested gender, we would add the gender tag accordingly. Otherwise, when the family relationship keyword (eg, spouse and parent) did not tell the gender, we used an external human name knowledge base to match the most likely gender with names. For instance, “Tom” and “Emily” indicated male and female, separately.

After constructing the GKGs from each obituary by modules 1 to 5, we assembled the extracted GKGs into bigger ones by matching PII, including people’s names, residence, birth date, death date, and family relationship.

#### Joint Training Loss

We minimized the negative log likelihood loss of the generated tags for the first 4 modules (module 5 is a rule-based inference layer that did not require training). For module *k* (*k*=1, 2, 3, 4), the loss function was defined as follows:


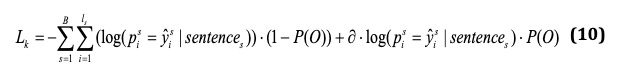


Where *B* was the batch size, *l_s_* was the length of input sentence sentences, *y^s^_i_* and *p^s^_i_* were the true tag and the normalized probability of the predicted tag for an input token *I*, and 𝜕 was a hyperparameter. *P(O)* was the indicator function that determined which part of equation 10 was used to calculate the loss. If the current tag was not “O” (other), the hyperparameter 𝜕 would decide the weight of the loss function. It was defined as follows:


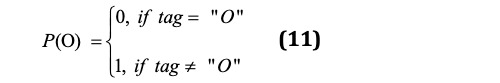


In the end, we combined all four loss functions *L_1_*, *L_2_*, *L_3_* and *L_4_* together, using different weighting parameters *λ_k_* into the final loss function, which was optimized for the entire training as follows:


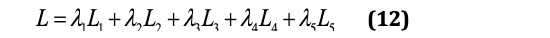


#### Evaluation Metrics

We performed 10-fold cross-validation by randomly selecting 10% of the annotated data for validation and the remaining for training. It is worth noting that the augmented data were only used for training models. Extracted GKGs consists of outputs from modules 1 to 5. They were measured by averaged performance of all modules except module 5 due to this rule-based inference module lacking a gold standard. For modules 1 to 4, we used precision, recall, and F-1 measure for evaluation, which were computed as follows:


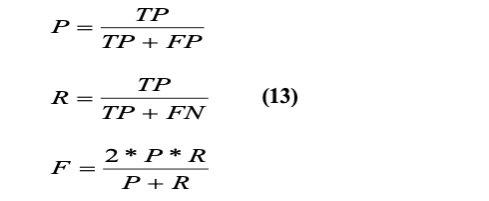


In module 1, the outputs were entity mentions with extra entity and relation types. We defined an extracted mention as true positive instances only if the mention’s boundary, entity type, and relation tags were exactly matched with the gold annotation. The instances of false positive were predicted mentions that do not precisely match with gold annotation boundaries, entity, or relation types. False negative instances were those existing in the gold annotation but not recognized by the model.

In module 2, true positive instances were defined as pairs of name and location that matched exactly. If either name or location was wrong, the pair would be considered a false positive. False negative referred to the name-location pairs missed by our system.

Module 3 and module 4 were formulated as generic classification tasks, so we used common definitions of false negative, false positive, and true positive. For all modules, evaluation metrics were precision, recall, and F-1 measure.

## Results

Table 3 illustrates the performance of modules 1 to 4 with ablation experiments in terms of macroaveraged and microaveraged precision, recall, and F-1 measure (without data augmentation). A macroaverage is the arithmetic average of the computed metrics for all classes and a microaverage sums up all true positive, true negative, false positive, and false negative instances before computing the final precision, recall, or F-1 measure for all classes. Macroaveraged metrics are often used for evaluation, particularly when there are extremely imbalanced classes, as no single class should largely dominate the results.

As shown in Table 3, we can see that module 1, which constructed the nodes and edges of the GKGs, achieved macroaveraged precision, recall, and F-1 measure of 83.85%, 83.05%, and 83.44%, respectively (see the third row of the macroaveraged performance). If we did not consider the effects of imbalanced data, the microaveraged precision, recall, and F-1 measure were even better, reaching 95.42%, 93.52%, and 94.46%, respectively (see the third row of the microaveraged performance). The macroaveraged results were much worse than the microaveraged results because the dominating classes had above-average performance. The minority classes, although having below-average performance, did not affect the microaveraged results much due to their small count numbers. For example, uncle-in-law, granduncle, and cousin-in-law had just 1 case in our corpus (see Figure 1). These relations affected the macroaveraged metrics more negatively. The performances for modules 2, 3, and 4 had similar patterns.

We also observed the benefits of multitask models through ablation experiments. Extra information gained from modules 3 and 4 seemed to improve module 1 in both macroaveraged precision, recall, and F-1 measure (2.17%, 3.12%, 2.64%, respectively) and microaveraged precision, recall, and F-1 measure (1.27%, 1.12%, and 1.19%, respectively). Modules 1 and 3 improved the performance of module 4 by 2.76%, 1.32%, 2.08% for macroaveraged precision, recall, and F-1 measure, respectively, and 2.51%, 1.7%, 2.13% for microaveraged precision, recall, and F-1 measure, respectively. Similarly, modules 1 and 4 helped to improve the macro/micro precision, recall, and F-1 of module 3 by 2.74%, 1.00%, 1.88%, respectively. And modules 1, 3, and 4 improved module 2 by 1.10%, 5.17%, and 3.49% in macro/micro averaged precision, recall, and F-1 measure.

It should be noticed that module 2 seemed not helpful in improving the overall performance of each module. For module 1, the macroaveraged and microaveraged F-1 measure dropped by 1.41% (compare the first and third row of the macroaverage section of Table 3) and 1.03% (compare the third and third row of the microaverage section) after introducing module 2 into the end-to-end system . Other modules had similar effects when included in module 2. This phenomenon was named negative transfer. It meant that although module 2 significantly benefited (F-1 measure raised from 75.08% to 78.57%), other modules were negatively affected. Liu et al [24] and Wang et al [25] also observed and discussed similar negative transfer effects. We talk about negative transfer further in the Discussion section. In our system, the solution for avoiding the negative transfer was that module 1, 3, 4 would be cotrained and module 2 would be separated from the whole system for training. In such a way, each module could benefit the most from the joint training method.

**Table 3:** Model performance of each module with ablation experiments.

Module and ablation test		Macroaveraged performance			Microaveraged performance		
		P^a^ (%)	R^b^ (%)	F1^c^ (%)	P (%)	R (%)	F1(%)
**Module 1**							
	Baseline	81.68	79.93	80.80	94.15	92.40	93.27
	Joint training (module 2, 3, & 4) + negative transfer	82.07	81.99	82.03	94.08	92.79	93.43
	Joint training (module 3 & 4)	83.85	83.05	83.44	95.42	93.52	94.46
**Module 2**							
	Baseline	83.17	68.43	75.08	—^d^	—	—
	Joint training (module 1, 3, & 4)	84.27	73.60	78.57	—	—	—
**Module 3**							
	Baseline	89.64	92.01	90.81	—	—	—
	Joint training (module 1, 2, & 4) + negative transfer	91.48	91.12	91.30	—	—	—
	Joint training (module 1 & 4)	92.38	93.01	92.69	—	—	—
**Module 4**							
	Baseline	90.65	94.74	92.64	90.96	95.21	93.03
	Joint training (module 1, 2, & 3) + negative transfer	92.34	95.76	94.02	92.37	96.31	94.30
	Joint training (module 1 & 3)	93.41	96.06	94.72	93.47	96.91	95.16

^a^P: precision.

^b^R: recall.

^c^F1: F-1 measure.

^d^The microaveraged and macroaveraged performances are the same for module 2 and module 3 because they are both binary classification tasks. All results shown are from the curated corpus without data augmentation.

**Figure 4:**
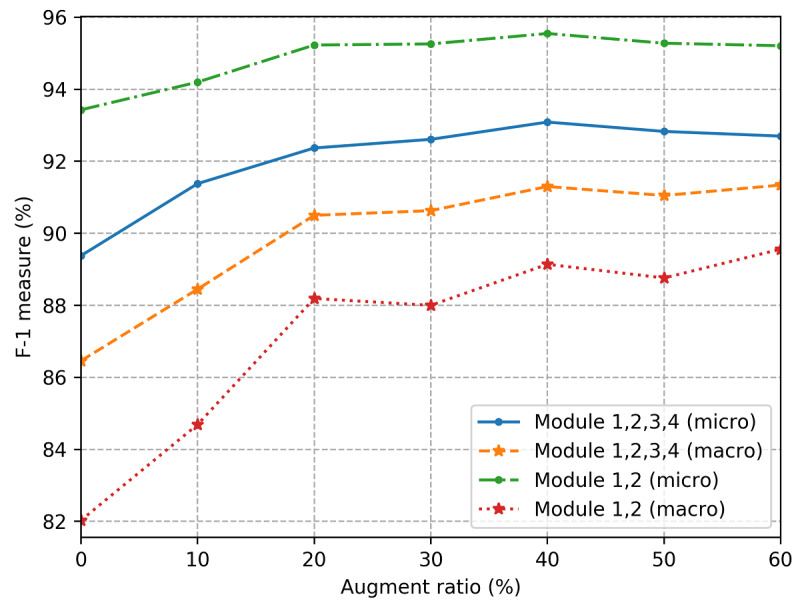
Comparing the F-1 measures of raw corpus and augmented corpus.

We also adopted data augmentation technology to expand our corpus, aiming to improve the relation extraction performance for family relations (module 1) with too few training examples. By synonym replacement, random insertion, random swap, and random deletion, we augmented the training data to ensure every relation had no less than 200 training examples. However, the automated data augmentation method introduced new noise. We tested a different augmentation ratio (*α*) to find the best balance. As shown in Figure 4, when the augmentation ratio was set to 40%, the extra synthetic data in training benefited our model most. It was worth noting that the augmentation data were only used in training for module 1, and we still evaluated our system with real, nonsynthetic test data. Figure 4 shown that the best macroaveraged and microaveraged F-1 measures achieved 89.14% and 95.55%, respectively, for module 1. With augmented module 1, our whole system achieved the best macroaveraged performances, 92.59% (precision), 90.05% (recall), and 91.30% (F-1 measure), and the best microaveraged metrics were 94.79% (precision), 91.45% (recall), and 93.09% (F-1 measure). These results confirmed that data augmentation technology can alleviate the problem of imbalanced data.

After extracting GKGs from all obituaries, we assembled them into bigger ones by matching available PII, including name, gender, age, residence, and birth date. Considering obituaries usually provide detailed PII for the deceased but not for their family members and relatives, we did fuzzy matching for the relatives. That is, if the mentioning of 2 people in 2 different obituaries are likely to refer to the same person based on 1 or more shared piece of PII, we would assemble 2 GKGs into 1. In the end, we had 319 GKGs assembled into 149 bigger GKGs after processing all 12,407 downloaded obituaries. Among those 319 obituaries, 22.3% (71/319) had 1 shared PII item, 8.5% (27/319) had 2, and 69.3% (221/319) had more than 2. We manually evaluated those 149 assembled GKGs and confirmed that 71.8% (107/149) were correct, 12.1% (18/149) were wrong, and 16.1% (24/149) were uncertain. We acknowledge that this rule-based matching method is limitedly useful for the selected geographic location of the Twin Cities area in Minnesota. It might be more error prone to apply to the entire country or other densely populated areas with high population mobility. So we did not include the assembly function in the end-to-end system but kept it as an additional resource for cautious users.

**Figure 5:**
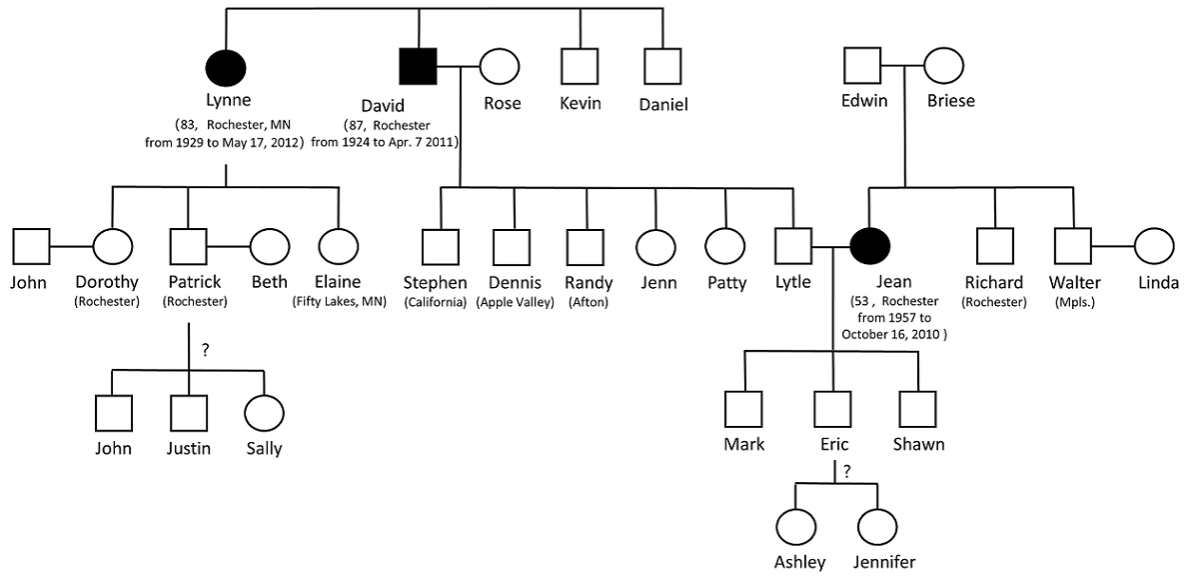
An example of an assembled genealogical knowledge graph. We removed last names for privacy protection. The symbol ? means we are not sure which children nodes belong to which parent nodes.

**Figure 6:**
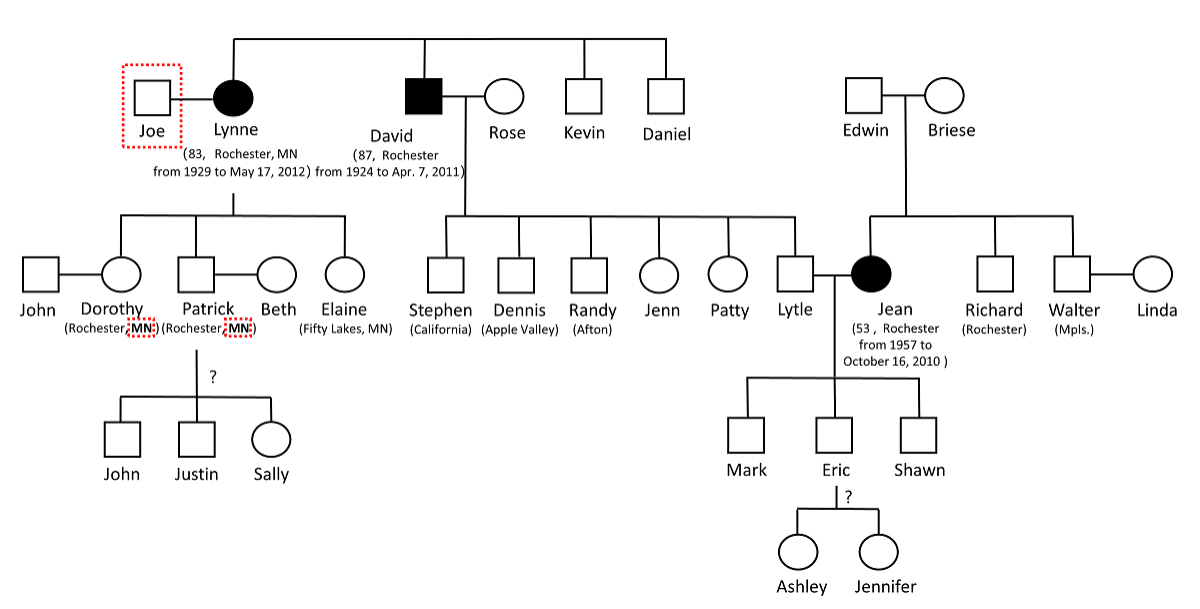
The gold standard family tree constructed from manual curation corresponding to Figure 5.

Figure 5 shows one example of assembled GKG from 3 obituaries. It contained 4 generations and 30 people. Figure 6 is the corresponding gold standard result conducted from manual validation. It can be seen that the assembled GKG missed the state name Minnesota for Dorothy and Patrick’s residences and one family member, Joe, who was Lynne’s husband (missing parts are shown in dashed boxes). In the original obituary, the sentence mentioning Lynne and Joe’s relation was “...he proposed...they began 54 years of happy life,” and our system failed to capture this subtle language. The successful assembly of multiple obituaries also demonstrated the feasibility of linking family relations extracted from obituaries to EHRs to support genetic research like linkage analysis and disease risk prediction. Meanwhile, it should be noticed that even though obituaries inherently contained rich genealogical information and the system extracts the GKGs with high accuracy, the GKGs should not always be equated to pedigrees used by genealogists. Although it is common to declare blood or nonblood relationships in obituaries due to data specialty (detail analysis for the slippery slope of genealogy issue shown in the Discussion section), we cannot guarantee people always declare the difference of blood or nonblood and always list all of their family members for various reasons.

## Discussion

### Principal Findings

In this work, we proposed an end-to-end system to construct GKGs from online obituaries, aiming at supplementing EHR data for genetic research. This system achieves microaveraged precision of 94.79%, recall of 91.45%, and F-1 measure of 93.09% after data augmentation technology. The work exploits the large availability of obituaries on the internet, which are consistent with the vital records and census records and more reliable and comprehensive than dependent information from medical insurance and emergency contact in EHR systems [5,6]. We demonstrate an efficient system to automatically build large GKGs from 10 years’ online obituaries in the Twin City area, Minnesota. Furthermore, by identifying individuals, we explore integrating related GKGs into bigger GKGs and manually validating the integrated results. The results show the feasibility of identifying individuals by extracted information, including residence, age, gender, birth, and death dates. We compute similarities between GKGs to further merge them into more complete GKGs. In the future, the similarity computing techniques could assist mapping the GKGs to the EHRs.

In this work, we use publicly available obituaries. The Association of Internet Researchers, in partnership with their Ethical Working Committee, formulated general principals to guide online research [26]. While this document presents the overarching ethical considerations relevant to social media–based research, a comprehensive determination of ethical principles and best practices has yet to be developed. Furthermore, debate continues as to whether some forms of social media–based research, namely analysis of existing textual archives (strictly speaking, online obituaries are not social media, but they have similar characteristics as a data source for biomedical research), fall within the parameters of human subject research or constitute an alternative form of humanistic inquiry [27]. Considering the PII embedded in online obituaries, we decided to take a cautious and conservative position in our work by marking up the last name of any real people mentioned in the paper.

As a novel data source, obituaries are informative for constructing family trees. It is hard to obtain such rich genealogical information from other data sources, but there are caveats to their use as genealogical data. First, semantic ambiguity occurs in obituaries as it occurs in many other types of human writing. For example, it is not uncommon to see statements like “...survived by two sons, Marshal and Paul XX and daughter Daisy, and four grandchildren Denny, Gary, Cecil, and Alina.” In this case, it is impossible to tell the exact parents for each of the 4 grandchildren Denny, Gary, Cecil, and Alina. All we know is that their parents are Marshal XX, Paul XX, and Daisy. Additional data sources like birth certificate registries can be helpful in this case.

A second point worth discussing is the slippery slope of genealogy. Compared with medical insurance and emergency contact information [5,6], a statement of nonblood relationship is more common in obituary data due to their specificity. As shown in Figure 1, for child relationship the ratio for nonblood versus blood is 483:5472 (there are 25 mentions of child-in-law, 99 of daughter-in-law, 105 of son-in-law, 151 of stepchild, 48 of stepdaughter, and 55 of stepson compared with 2489 cases of child, 1399 of daughter, and 1584 of son). A similar ratio can be observed in nonblood parent relationship. This advantage could be helpful for alleviating the problem of the slippery slope of genealogy. However, it is still worth mentioning that not all people make such distinctions in obituaries.

**Figure 7:**
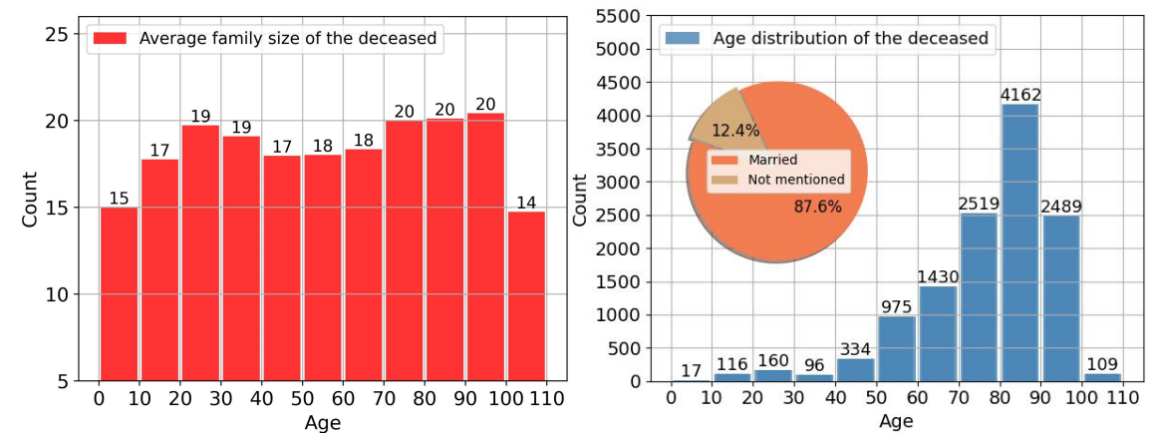
Left: distribution of average numbers of mentioned family members. Right: age and marital status of the deceased person in 12,407 extracted genealogical knowledge graphs.

In addition, Figure 7 displayed the related statistics aimed at showing potential data bias. We plotted the distribution of age (at death), average number of mentioned family members, and marital status of the deceased for all GKGs extracted from 12,407 downloaded obituaries. As shown in Figure 7, the age distribution of the deceased is consistent with public health data (73.9% of the deceased died at the ages of 70 to 100 years). The average numbers of mentioned family members seem similar for different age groups; only those died in the 0 to 10 and 100 to 110 age groups had relatively smaller family size (≤15); 87.6% of the GKGs indicated that the deceased was married at least once. We did not interpret the results too deeply because we did not have a good understanding of the sample bias. Meanwhile, it was noticed that people who had complete and/or affluent families tended to publish obituaries. Although these data biases would not affect the performance of our extraction system, the fact that extracted GKGs may be biased should be considered when researchers are using them in other research.

Technically, the data used in the research are very imbalanced, in which 14 rare relationships have fewer than 10 instances. We adopted the augmentation technology to enhance system performance. For example, in the relationships half-sister, grandchild-in-law, and grandson-in-law, their F-1 measures increased from 20.0%, 30.0%, and 35.71% to 66.67%, 50.0%, and 71.43%, respectively. Next step, we plan to experiment with additional few-shot (extremely imbalanced)–based information extraction and mate learning to improve the system [28,29].

In our end-to-end solution, the performance of module 2 was obviously inferior to the other modules. Besides the error propagation problem (module 2 need the results from module 1), the task of module 2 was a semantic matching resolution problem, which is still challenging in the NLP community. In addition, we currently have curated an obituary corpus in English to train the neural network models. To expand to other languages, a new corpus in those specific languages and new gender inference rules would need to be curated. There is some cross-language transfer research in the NLP community which suggests neural models trained on an English corpus can help to build NLP models in other languages by reducing training data and training time. Sometimes such transfers even provide more robust models with better performance [30,31].

In our end-to-end solution, module 2 currently is the bottleneck. This module suffered significantly from negative transfer. Generally speaking, when a task or domain was joined with data of no relatedness or similarity, the added data would become noise rather than useful information. It remains challenging to quantitatively measure the relatedness or similarity among different tasks or domains [32]. Therefore, most transfer learning solutions rely on empirical methods and do not account for negative transfer effects. In this work, we considered module 2, which matched locations to people, as strongly related to other modules that extracted locations or people and paired them. Unfortunately, the experiment results showed negative transfer still occurred. One possible explanation was about the different natures of tasks in modules 1, 2, 3, and 4. Module 2 was a classification task with 2 entity mentions as the input and a class tag as the output. All other modules were sequence tagging tasks, where the whole sentence was the input and tags for all tokens of an input sentence were output. Another possible reason was that the task of module 2 was much more challenging than the others. Modules 1, 3, and 4 all had a higher than 90% microaveraged F-1 measure when we tested them individually, while module 2 had a 75.08% microaveraged F-1 measure. In addition, module 2 needed inputs from module 1. The errors of module 1 would propagate to module 2. How to improve module 2 and alleviate its negative transfer and error propagation is what we plan to focus on methodologically in the future.

Besides the performance benefits shown in the Result section, the multitask solution is also faster to train. We use a single V100 GPU in this study. For the traditional pipeline model, one round 10-fold cross-validation experiment costs about 240 hours in total. However, the multitask model with all 4 modules together takes only 150 hours. For module 1, the training process took about 70 epochs to achieve an F-1 measure of 80% when being trained independently. The multitask method takes less than 5 epochs to achieve the same level of F-1 measure.

### Limitations

The first limitation of our work is the existing potential data bias. Our data are collected from online obituary websites. In such conditions, people who had intact and/or affluent families tended to publish obituaries. The second limitation is that our system is mainly for English obituaries. Modules 2 and 3 are designed for 2 English writing patterns.

### Conclusions

GKGs have great potential to enhance many medical research fields, especially combined with EHR data. We believe a high-quality, large-scale genealogical information database will have significant research meaning. In this work, we presented a new corpus with a predefined family relationship map and augmented training data and proposed a multitask deep neural system to construct and assemble GKGs. With the data augmentation technology, the system achieved microaveraged precision, recall, and F-1 measure of 94.79%, 91.45%, and 93.09%, respectively, and macroaveraged precision, recall, and F-1 measure of 92.59%, 90.05%, 91.30%, respectively. Based on such promising results, we developed PII-matching rules to assemble large GKGs, demonstrating the potential of linking GKGs to EHRs. The system is capable of generating a large number of GKGs to support related research, like genetic research, linkage analysis, and disease risk prediction. We share the source codes and system with the entire scientific community on GitHub, without the corpus for privacy protection [33].

In the future, we will improve the performance of our system further and match GKGs with more medical information, like EHR databases. With the massive obituary data freely available on the internet or other textual data that contain genealogical information, our ultimate goal is to accelerate large-scale disease heritability research and clinical genetics research.

## Abbreviations

EHR: electronic health records
GKG: genealogical knowledge graph
LSTM: long short-term memory
NER: name entity recognition
NLP: natural language processing
PII: personally identifiable information
RC: relation classification
